# Pharmacokinetics of Vancomycin among Patients with Chemotherapy-Associated Febrile Neutropenia: Which Would Be the Best Dosing to Obtain Appropriate Exposure?

**DOI:** 10.3390/antibiotics11111523

**Published:** 2022-11-01

**Authors:** Daniel Parra González, Jefferson Alejandro Pérez Mesa, Sonia Isabel Cuervo Maldonado, Jorge Augusto Díaz Rojas, Jorge Alberto Cortés, Edelberto Silva Gómez, Carlos Humberto Saavedra Trujillo, Julio Gómez

**Affiliations:** 1 Department of Pharmacy, Faculty of Sciences, Universidad Nacional de Colombia, Bogotá 111321, Colombia; 2 Faculty of Medicine, Universidad Nacional de Colombia, Bogotá 111321, Colombia; 3 Grupo de Investigación en Enfermedades Infecciosas en Cáncer y Alteraciones Hematológicas (GREICAH), Universidad Nacional de Colombia, Bogotá 111321, Colombia; 4 Instituto Nacional de Cancerología (INC)—Empresa Social del Estado, Bogotá 111511, Colombia; 5 Group on Research in Infectious Diseases, Faculty of Medicine, Universidad Nacional de Colombia, Bogotá 111321, Colombia

**Keywords:** pharmacokinetics, vancomycin, chemotherapy-induced febrile neutropenia, hematologic neoplasms, therapeutic drug monitoring, area under curve, minimum inhibitory concentration

## Abstract

Previous research has determined that the required doses for treating febrile neutropenia with vancomycin are higher than the doses used conventionally. These recommendations have been made considering pharmacotherapeutic goals based on minimum concentration ($C_{\min}$) between 15–20 mg/L. This study was developed to evaluate dose recommendations based on the achievement of a target consisting of ratio of area under the curve over minimum inhibitory concentration (${\mathrm{AUC}}_{24h}/\mathrm{MIC}$) ≥400 in this population of individuals. This study was conducted in a referral hospital for cancer treatment, study participants received vancomycin doses of 1g every 12 h in 2-4-h infusions. Vancomycin was described by a two-compartment pharmacokinetic model with clearance dependent on the estimated glomerular filtration rate. Simulations were performed taking into account a reduced version of the model to establish the influence of controllable and non-controllable variables on the probability of achieving several PK-PD targets. A dose of 2.5g/day in patients with estimated glomerular filtration rate (eGFR) between 80 and $122\;\mathrm{mL}/\min/1.73\;m^{2}$ was adequate to achieve the pharmacotherapeutic target. A discrepancy was found between AUC-based and $C_{\min}$-based PK/PD indices, the former being affected by the dose and creatinine clearance while the latter highly influenced by the interval between doses.

## 1. Introduction

Infection in patients with chemotherapy-induced neutropenia is extremely frequent; 80% of patients may have a febrile episode, and about half undergo an infectious process, between 15% and 38% of the cases identified are produced by Gram-positive cocci [1,2]. Although mortality in patients with Gram-positive bacterial infections is between 2% and 12% [3,4], rapid and effective antimicrobial therapy can reduce hospital stays, complications, and fatal outcomes [5].

Vancomycin (VAN) is an antibiotic useful in treating Gram-positive bacterial infections such as *Methicillin-resistant Staphylococcus aureus* [6]. VAN is used, under certain conditions, in the empirical treatment of febrile neutropenia [7].

The association betwee pharmacokinetic/pharmacodynamic (PK/PD) indices of VAN and clinical effectiveness (e.g., reduction in mortality or minimization of therapeutic failure) has been evaluated mainly by observational studies [8]. Levels of AUC/MIC≥400 with VAN treatment have been associated with positive outcomes such as decreased therapeutic failure and increased mortality [9,10,11,12]. On the other hand, high levels of area under the curve (AUC) (e.g., higher than 650 mg·h/L) have been associated with an increased risk of acute kidney injury (AKI) [13].

In 2020, an update of the guidelines for the treatment of *S. aureus* infections with VAN was published [6]. In these guides, the recommendation of the objective was made AUC/MIC≥400 as the main predictor of success with VAN therapy which contrasts with the previous version of this guide, where it was recommended to $C_{\min}$ between 15–20mg/L as the main objective [14]. Recently, results have been found indicating that monitoring of VAN with $C_{\min}$-based targets may lead to an increase in the incidence of VAN-induced kidney failure compared to AUC-based targets [15,16], although a correlation between these PK/PD indices has been reported [17,18]. Implementing this recommended change may have obstacles because it implies a shift in the prescription culture and additional technological requirements for the estimation of AUC [19].

Although there are several PK models published in the literature for VAN in cancer patients [20,21,22,23,24] the simulations for dose selection have been based only on $C_{\min}$-based PK/PD indices. This study aimed to describe the behavior of VAN plasma concentrations determined by a chemiluminescence based method in patients with post-chemotherapy febrile neutropenia. The developed model was used to find appropriate dosing schemes (by means of population simulation) for achieving PK/PD targets and finally to understand the impact of the variables on the probability of achieving pharmacotherapeutic targets.

## 2. Materials and Methods

### 2.1. Study Design and Ethics

An open, observational, descriptive, prospective, and non-randomized study was conducted in the hematology service of the Instituto Nacional del Cancerología (INC), the first public hospital in Colombia that has 180 beds to treat cancer patients from all over the country and located in Bogotá, Colombia. The study was carried out with prior approval of the protocols by the Research Ethics Committee of the INC and the Faculty of Sciences of the Universidad Nacional de Colombia. Informed consent was obtained for all study participants.

Inclusion criteria were: (i) patients over 18 years of age, (ii) diagnosis of confirmed hematologic neoplasia, (iii) treatment with chemotherapy induction, maintenance, or rescue, (iv) neutropenia defined by an absolute neutrophil count (ANC) less than 1000 μL, (v) fever defined as a single oral temperature $\geq 38.3{\;}^{\circ }C$ or a temperature $\geq 38.0{\;}^{\circ }C$ sustained for 1 h [25], and (vi) treatment with single VAN or in combination with another antimicrobial.

Exclusion criteria were: (i) acute kidney failure [26], (ii) chronic kidney disease defined by an estimated glomerular filtration rate (eGFR) less than $60\mathrm{mL}/\min/1.73\;m^{2}$ [27], (iii) hepatic failure defined by Child-Pugh B or C [28], altered liver morphology or architecture, fibrosis or necroinflammation, (iv) significant comorbidities other than cancer, (v) diabetes mellitus, (vi) congestive heart failure, (vii) hypothyroidism, and (viii) pregnancy. The eGFR was calculated using the CKD-EPI equation [27].

### 2.2. Administration and Determination of Concentrations

VAN (Brand Vitalis, Barranquilla, Colombia) was administered intravenously at a dose of 1 g every 12 h in infusions of 2 to 4 h. 5 mL of venous blood was taken via a dedicated catheter for sampling 48 h after initiation of therapy. In total, 7 blood samples were taken at 1, 2, 4, 6, 8, and 10 h after the start of the infusion and 30 minutes before the next VAN dose.

Samples were collected in a blood chemistry tube BD Vacutainer reference 367986 and were processed with centrifugation at 3500 rpm for 10 minutes. The supernatant serum was transferred to cryovials and stored at $-70{\;}^{\circ }C$ until the time of analysis.

Measurement of VAN concentrations were carried out by determination with a valitated chemiluminescence-based method [29]. The chemiluminescence immunoassay was applied on the Architect i1000R equipment (Abbot Diagnostics) and the iVancomycin kit (Abbot diagnostics) designed for pharmacotherapeutic monitoring of VAN. The chemiluminescence method was calibrated by using samples with known concentrations following the manufacturer’s instructions, a linearity range between 20–80 μg/mL ($R^{2}=0.9968$) was observed with a 13.7% accuracy and a lower limit of quantification of 3 μg/mL.

### 2.3. Pharmacokinetic Modeling

A population pharmacokinetics model for VAN was developed using the Expectation-Maximization with Stochastic Approach (SAEM) algorithm with Monolix 2019-R2 software (Lixoft SAS, Antony, France) [30]. For the base model, structural models of one, two, or three compartments with linear elimination were evaluated, as well as several models for the residual error component (additive, proportional or combined). The residual error model was specified as assuming a lognormal distribution (see Equation 1 for the additive form of the model).

(1) $$\ln y_{ij}=\ln f\left(\beta_{i},x_{i},t_{ij}\right)\cdot \left(1+b\varepsilon_{ij}\right)$$

Wherein, $f\left(\beta_{i},x_{i},t_{ij}\right)$ is the prediction of the model according to the pharmacokinetic parameters of each individual ($\beta_{i}$), the dosing regimen ($x_{i}$), and the times ($t_{ij}$) at which the determinations are made. $\varepsilon_{ij}$ is a random variable with a standard normal behavior $N\left(0,1\right)$, while *b* refers to the proportional parameter for residual variability. The parameters of each individual *i* ($\beta_{i}$) were estimated with an exponential equation $\beta_{i}=\theta \cdot \exp\left(\eta_{i}\right)$, in which θ is a population parameter and $\eta_{i}$ is the deviation of the parameter with a distribution (0, $\omega^{2}$). The likelihood ratio test (LRT) and Bayesian information criterion (BIC) [31] were used to test various hypotheses related to structural model selection, covariates, and error.

For the construction of the final model, a series of covariates were selected from a set of available variables by screening with generalized additive models (GAM). The selected covariates were sex, total body weight, age, height, serum creatinine ($S_{\mathrm{CR}}$), eGFR, and serum protein concentration. These covariates were evaluated due to their possible effect on the pharmacokinetics of vancomycin according to previous reports [32] or their biological plausibility in their relationship with VAN’s pharmacokinetics. The inclusion of the covariates was performed by linear, potential, or exponential equations for continuous covariates and by additive equations for discrete covariates. In the incorporation of continuous covariates, these were scaled on the sample median. The selection of the covariates was guided by the application of the COSSAC algorithm [33], taking into consideration a decrease in the objective function of −3.84 (p<0.05) for forwarding inclusion and a change of +6.63 (p<0.01) for backward elimination. The covariates of the final model were determined by the improvement of the goodness-of-fit of the model, as well as by the biological plausibility of the relationship between covariates and pharmacokinetic parameters.

Goodness-of-fit was evaluated graphically; similarly, the distributions of the residuals were evaluated. Predictive performance measures such as mean absolute error (MAE), mean absolute error percentage (MAPE), and root mean quadratic error (RMSE) were calculated for individual and population predictions. An internal model validation was performed using a prediction-corrected visual predictive check (pcVPC) for each determination method [34]. The pcVPC was constructed by simulating the study design 1000 times in the interval 0–12 h after the administration of the dose. Empirical intervals were obtained 80% from 7 bins of equivalent size formed with the observations. The empirical intervals were compared with the prediction intervals of 80% and confidence bands of 95% obtained from the simulated data.

### 2.4. Pharmacodynamics Assessment/Simulations

The evaluation of VAN pharmacodynamic behavior for this patient population was performed, taking into account the final constructed model and several PK/PD indices. A primary PK/PD index was the achievement of the PK/PD target of ${\mathrm{AUC}}_{24h}/\mathrm{MIC}\geq 400$ over a 24-h time interval [6]. Two other secondary indices characterized by an upper limit were also considered to reduce the risks of adverse drug reactions associated with VAN of (i) ${\mathrm{AUC}}_{24h}$ between 400–600 mg·h/L and (ii) $C_{\min}^{\mathrm{SS}}$ between 15–20 mg/L.

The PK/PD indices were simulated at a steady state over a period of 72 to 96 h after the start of the dosing regimen. For the generation of simulations and the evaluation of exposure PK/PD indices, the R mlxR package [35] was used. We explored the effect of controllable variables related to the dosing regimen, such as daily dose (DD), interval between doses (II), and infusion times ($T_{\inf}$), and not controllable as minimum inhibitory concentration (MIC) or eGFR in the scope of the pharmacotherapeutic targets determined by the probability of target attainment (PTA), which was considered optimal if at least 0.90 was reached according to the dose recommendations [36]. To calculate the area under the curve (AUC), the log-trapezoidal method was used at the defined time intervals.

The effect of the variables on PTA was explored by reducing the model through the implementation of a pre-fed artificial neural network (ANN). The study of the achievement of PK/PD indices was carried out by training two different neural networks from simulated data with the final model of VAN [37]. The ANNs were specified and trained through the TensorFlow library [38] with its API in Python 3.7. The use of two ANNs was necessary because the secondary PK/PD indices had 4 variables (DD, $T_{\inf}$, II, eGFR), but the primary indicator had an additional variable ($\log_{2}\mathrm{MIC}$).

**Figure 1:**
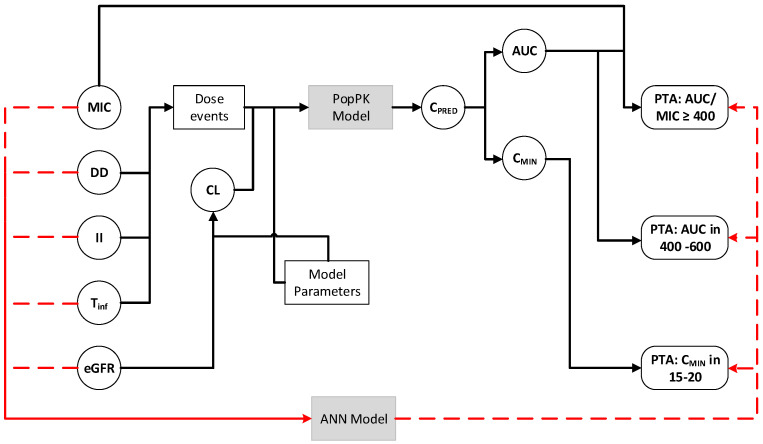
Schematic representation of the applied model reduction.

In the model specification, 4 different neural networks architectures (see Figure 1 general model) were evaluated with the following specifications:

- Preprocessing layer with normalization of input variables.
- Input layer with 4 to 5 nodes according to the type of index with rectified linear units (ReLUs).
- Hidden layer 1 with 5 (network A) or 10 nodes (networks B, C, and D) with ReLUs.
- Hidden layer 2 with 10 nodes (C and D networks), with ReLUs.
- Hidden layer 3 with 10 nodes (network D only), with ReLUs.
- Output layer with a sigmoid activation function (PTA only defined between 0 and 1). The ANN for prediction of the AUC/MIC-based index contain only one node in this layer, while the ANN for secondaryindices was considered as a multi-output model with two nodes in the output layer.

For the training of the model, simulations were carried out with the final model and various combinations of the input variables taking into account the following continuous uniform distributions: (i) $\mathrm{DD}∼U\left(1000,4000\right)$, (ii) $\mathrm{eGFR}∼U\left(80,150\right)$, (iii) $\mathrm{II}∼U\left(2,24\right)$, (iv) $T_{\inf}∼U\left(1.5,II\right)$, and (v) $\log_{2}\mathrm{MIC}∼U\left(-2,2\right)$. 3000 different dose regimens and conditions were created by combination of simulated values of DD, eGFR, II, $T_{\inf}$, and $\log_{2}\mathrm{MIC}$, and for the determination of exposure parameters (as well as PTA), 1000 individuals were simulated by regimen.

The simulated data were separated into a training (n = 2400) and test (n = 600) data set, and normalization of the data was applied considering the mean and standard deviation of the variables of the complete data set. Model training was performed with adaptive moment estimation (Adam) optimization [39], with MAE as the objective function, a batch size of 256, a maximum number of epochs for training of 1000, and scheduled early stops when no changes smaller than $1\cdot 10^{-10}$ were present in 30 epochs (patience). The model was selected according to the predictive performance on the test data set, considering the value of MAE and RSME, the models were evaluated graphically using loss curves, boxplots of residuals for training and test sets per model, and goodness-of-fit plots (results not shown). Trained ANNs were used to simulate outcomes for various dosing regimens and to explore the impact of controllable (DD, II, and $T_{\inf}$) and non-controllable (MIC or eGFR) variables. The impact of covariates was explored through changes in one to two variables while maintaining the rest of the variables with constant values.

The influence of the variables in predicting PTA (through the ANN) was evaluated by Kernel SHAP. Kernel SHAP was performed using the KernelExplainer function in the SHAP module (version 0.40.0) of Python [40] by randomly taking 400 data points sampled from the training data.

## 3. Results

### 3.1. Patient Characteristics

A total of 14 patients participated in the study, with 96 determinations of VAN concentration. Patients included in this study received VAN for the empirical treatment of post-chemotherapy febrile neutropenia in combination with other antibiotics such as cefepime, meropenem, or piperacillin/tazobactam. Hematologic malignancy was myeloid leukemia (n = 6), lymphoid leukemia (n = 5), and Non-Hodgkin’s lymphoma (n = 3). The demographic characteristics of the patients included in the study are presented in Table 1. The majority of patients received the first cycle of chemotherapy (42.9%).

### 3.2. Pharmacokinetic Analysis

**Figure 2:**
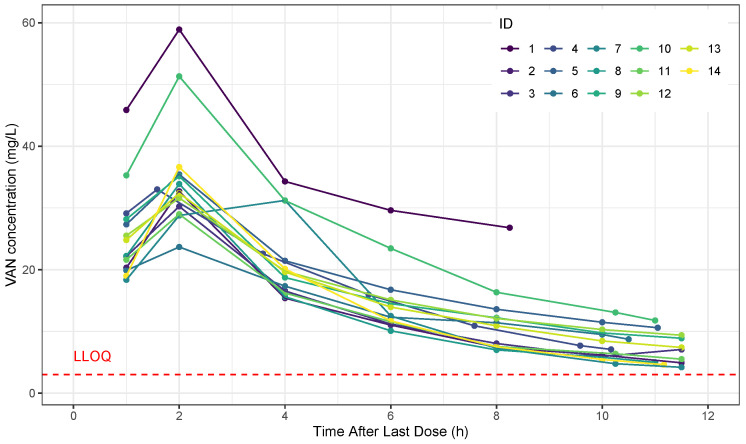
Observed plasma concentration vs Time After Last Dose profile for vancomycin.

VAN concentrations were found between 4.18 and 58.90 mg/L, with minimum concentrations between 4.18–26.79 mg/L and maximum concentrations between 23.67–58.90 mg/L for subjects. The Figure 2 show the relationship between the observed plasma vancomycin concentration and time after the dose. Table 2 summarizes the parameters estimated for the final model, as well as its structural and statistical components.

The final model presented with estimated glomerular filtration rate (eGFR) as one of the factors affecting VAN clearance (Cl), and eGFR was included in the form of a potential equation [41] and centered on the median (see Equation (2)).

(2) $${\mathrm{Cl}}_{i}=\theta_{0}\cdot {\left(\frac{\mathrm{eGFR}}{144}\right)}^{\theta_{1}}$$

Wherein $\theta_{0}$ is the VAN clearance value when eGFR is equal to $144\;\mathrm{mL}/\min/1.73\;m^{2}$, and $\theta_{1}$ reflects the change in Cl per unit centered of eGFR both in the logarithmic domain. A variance-covariance matrix for interindividual variability was used with a correlation (ρ) between the $V_{1}$ and parameters Cl. The residual error model was represented using an equation with a proportional error term.

**Figure 3:**
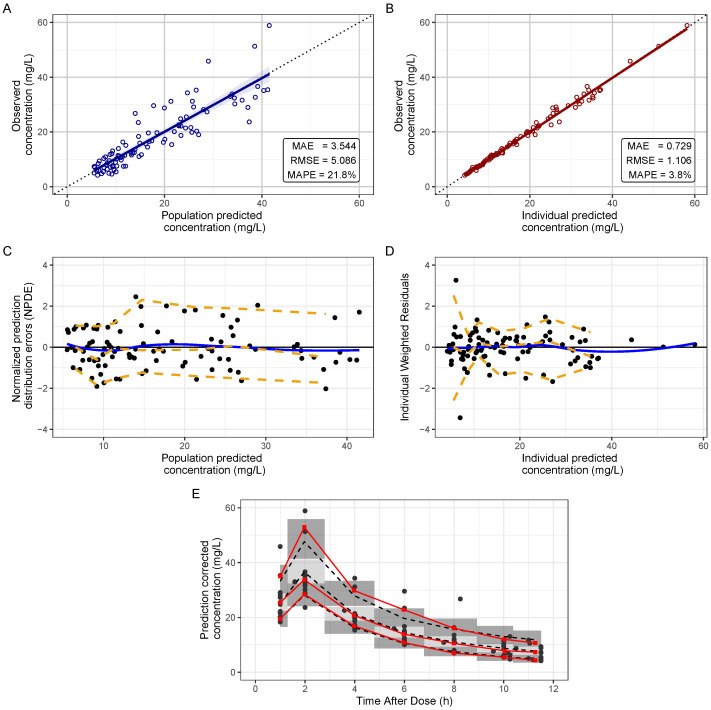
Goodness—of—Fit plots and visual predictive check for final VAN model. (**A**) Observation vs. population predictions plot for the final model. (**B**) Observation vs. individual predictions plot for the final model. (**C**) Normalized prediction distribution errors vs population prediction. (**D**) Individual Weighted Residuals vs Individual predicted concentration. (**E**) Prediction corrected visual predictive check (pcVPC) for each VAN concentration determination method, 2.5, 50, and 97.5% percentiles of observed data (solid red line) were shown, as well as simulated data (dark dash line). The confidence intervals for the percentiles of the simulated data are shown in the form of grey bands.

The goodness-of-fit plots for the final model (Figure 3A,B) do not show any systematic tendency for the final model with underprediction for observed concentration in one subject. Systematic bias is lower for individual predictions (Figure 3B). The final model had a low mean absolute percentage error (MAPE) with values of 21.8% and 3.8% for population and individual predictions respectively.

In the residual plots (Figure 3C,D), no systematic trends or changes in variance were observed according to variables such as time after dose administration (TAD) or concentration predicted by the model ($C_{\mathrm{PRED}}$). In Figure 3E, it is observed the final model’s prediction corrected visual predictive check (pcVPC). There is a good agreement between the intervals of prediction of the observed data and those of simulations of the final model, with differences only in high concentrations.

In the studied sample, an ${\mathrm{AUC}}_{0-12h}$ of 182.06 (158.82–202.09 mg·h/L was obtained for individuals that can be extrapolated to an ${\mathrm{AUC}}_{0-24h}$ of 364.12 (317.64–404.18) mg·h/L for a dosage of 1g of VAN every 12 h. Minimum steady-state concentrations ($C_{\min}^{\mathrm{SS}}$) were 7.24 (4.99–9.38) mg/L, on the other hand, the maximum steady-state concentrations ($C_{\max}^{\mathrm{SS}}$) were in 32.84 (31.21–35.42) mg/L. The exposure measures reported in this paragraph were expressed in median and interquartile ranges.

### 3.3. Pharmacodynamic Results

According to the exposure calculations obtained in the sample, 4 out of 14 individuals would have had levels of ${\mathrm{AUC}}_{0-24h}\geq 400\;$mg·h/L; for that reason, it is expected that the main pharmacotherapeutic target would not be fulfilled with the dose of 1 g q12 h in 2-h infusion in most of the patients. On the other hand, 3 of 14 individuals would present levels of ${\mathrm{AUC}}_{0-24h}$ between 400 and 600 mg·h/L, while none of the individuals presented a value of $C_{\min}$ in the range between 15–20 mg/L.

Table 3 shows the results of predictive performance indices for the training and testing datasets for the two trained ANNs. The error magnitude between test and training data was similar, suggesting that trained networks do not overfit. The error is more significant for model 2 compared to model 1 (see Table 3). This error is inherent to the PTA simulations (i.e., the probability is estimated accurately only when the number of subjects is very large) with which the model was trained.

**Figure 4:**
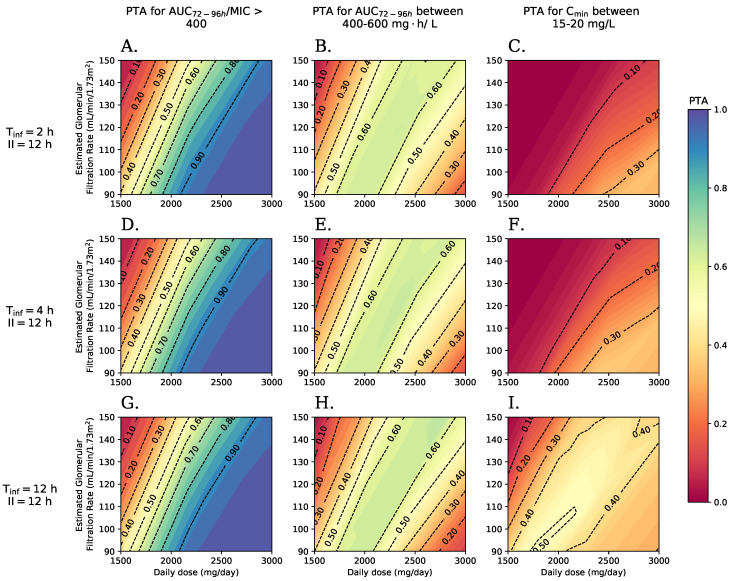
Response surface plots for PTA of explored PK/PD indices. (**A**,**D**,**G**) PTA for index ${\mathrm{AUC}}_{72-96h}/\mathrm{MIC}\geq 400$ for times of infusion of 2, 4, and 12 h respectively. (**B**,**E**,**H**) PTA for index ${\mathrm{AUC}}_{72-96h}$ between 400–600 mg·h/L for times of infusion of 2, 4, and 12 h respectively. (**C**,**F**,**I**) PTA for indicator $C_{\min}^{\mathrm{SS}}$ between 15–20 mg/L for times of infusion of 2, 4, and 12 h respectively. The evaluated VAN dosing schemes presented a 12-h period as II, $T_{\inf}$ with 2 h and MIC of 1mg/L only for the AUC/MIC index.

Figure 4 shows surface graphs showing PTA behavior for several indices with various VAN dosing regimens in the columns. The dosing schemes were constructed with various daily dose values (between 1500–3000 mg/day) with interdose interval of 12 h, various creatinine clearance values (90–150 $\mathrm{mL}/\min/1.73\;m^{2}$), and various infusion times in the rows with 2, 4 and 12 h for the top, middle and bottom row respectively. The PTA value for each index changes according to the dosing scheme. Dotted lines are shown with the outline of various regions according to the value of calculated PTA.

Panel A of Figure 4 shows the behavior of the main PK/PD index ${\mathrm{AUC}}_{24h}/\mathrm{MIC}>400$ by using a MIC value of 1mg/L to facilitate comparison with the rest of the indices. It was observed that PTA tends to increase with daily dose and to decrease with eGFR.

For ${\mathrm{AUC}}_{24h}/\mathrm{MIC}>400$, PTA values close to 1 are reached. Because of this, recommendations can be made for minimum daily dose levels according to the level eGFR determined for each patient. A daily dose of 2 g (1 g q12 h in infusion of 2 h) allows reaching PTA values of at least 0.90 in patients with eGFR less than $80.4\;\mathrm{mL}/\min/1.73\;m^{2}$, while a daily dose of 2.5 g (1.25 g q12 h in infusion of 2 h) allows reaching this threshold in patients with eGFR up to $122.4\;\mathrm{mL}/\min/1.73\;m^{2}$.

The value of MIC had a significant influence on the PTA for the objective of AUC/MIC≥400. It was observed that if the microorganism has a MIC value of 0.5mg/L, this resulted in a compliance of PTA close to 1 for all daily dose combinations and eGFR explored. On the other hand, a value of MIC of 2mg/L prevents the PK/PD target from being reached in almost all patients.

Panel B of Figure 4 shows the behavior of the index AUC between 400–600 mg·h/L. This graph shows a petal shape due to the upper limit on the PK/PD target. In this case, a PTA higher than 0.68 is not met for any daily dose combination and eGFR. The inability to achieve PTA high dose levels prevent the establishment of general dose recommendations without therapeutic drug monitoring (TDM). The recommended daily dose regarding the value of eGFR is similar to those obtained with the primary PK/PD index, with the need to use TDM to verify compliance with the PK/PD targets in patients.

Panel C of Figure 4 shows the behavior of PTA for the PK-PD pharmacotherapeutic target of $C_{\min}^{\mathrm{SS}}$ between 15–20 mg/L. This index shows the lowest values of PTA among the three indices explored, with maximum values of up to 0.35 only with high eGFR and low daily doses. This index shows a very different behavior to that observed with the indices based on AUC, so it may not function as a subrogate of them. The interval between doses and the infusion time has an important influence on this index since increasing $T_{\inf}$ or decreasing II improves the compliance index and can reach a level PTA of up to 0.5 (see Panel I of Figure 4).

**Figure 5:**
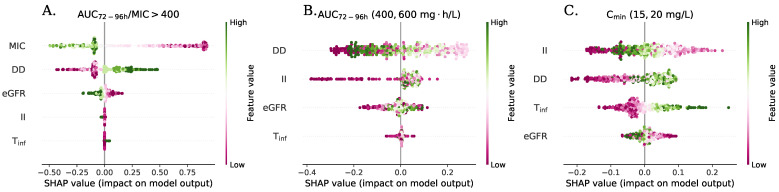
SHAP summary plots for the artificial neural network models created. (**A**) Index ${\mathrm{AUC}}_{72-96h}/\mathrm{MIC}\geq 400$. (**B**) Index ${\mathrm{AUC}}_{72-96h}$ between 400–600mg·h/L. (**C**) Index $C_{\min}^{\mathrm{SS}}$ between 15–20 mg/L.

Figure 5 shows scatter plots with the SHAP values for the ANN models developed. These values reflect the impact and importance of the variables on the result PTA obtained for a dosing regimen. The variables in each index are organized in descending order to reflect their importance in the predictions of PTA. For the index ≥400, the most important variables are MIC and daily dose (DD). An inversely proportional relationship was presented between MIC and PTA, while the opposite is true with DD. The eGFR showed less importance and had an inversely proportional relationship with PTA. The variables of $T_{\inf}$ and II did not exhibit high importance in the value of PTA for this index, so increasing the drug infusion time would not present an advantage.

Figure 5 Panel B shows the relative importance of the variables in the achievement of AUC between 400–600 mg·h/L. The importance of the variables was similar to that observed for the primary index, although II is more important than eGFR. It was observed that very high or low values of DD produced PTA low values, while intermediate values of DD generate higher PTA values. The variables II, $T_{\inf}$, and eGFR were inversely proportional to the value of PTA.

The index $C_{\min}^{\mathrm{SS}}$ between 15–20 mg/L showed a different behavior than that observed for the indices based on AUC. The variables associated with the intensity of administration were of equal or greater importance than the daily dose or eGFR. It is observed that PTA was favored by the use of intermediate values II and longer infusion times. The daily dose and eGFR presented a direct and inversely proportional correlation in a respective manner versus PTA.

## 4. Discussion

This pharmacokinetic study shows that the behavior of VAN in patients with post-chemotherapy febrile neutropenia is similar to that of healthy patients. The evaluated PK/PD models suggested that it is possible to achieve the expected parameters but that the proportion of patients in whom these objectives are achieved depends on the MIC, dosage, and renal function of the patient, which must be taken into account when prescribing this medication in this scenario.

A two-compartment model was found with a low relative standard error (RSE) in the parameters, the typical value of clearance (TVCl) of 5.2L/h being within the range described for a group of diverse adult populations (median, interquartile range—IQR) 3.22 (2.32–4.9) L/h [32]. This value Cl is similar to the reported value in a population of patients without febrile neutropenia with Cl de 4.90L/h [42]. The total volume of distribution was 49.5L ($V_{\mathrm{total}}=V_{1}+V_{2}$), which is lower than that reported for a group of various populations (median, IQR) 80.7 (47.8–97.15) L/h [32]. This $V_{\mathrm{total}}$ was similar to that of studies in patients without febrile neutropenia, such as 46.2L [42] and 46.3L [43].

An interindividual variability of clearance ($\omega_{\mathrm{Cl}}^{2}$) of 22.3% was found, which was similar to what is found in non-neutropenic patients, for example, 26.70% [42] and 20.8% [44].

Hirai K et al. [20] reported low values of Cl (2.82L/h) and high values of V (108L), assuming a weight of 60Kg. On the other hand, the study by Haeseker M. [21] reported a similar Cl (4.08L/h) and a higher V (62L) in comparison with this study. The findings are heterogeneous in different studies, Jarkowski A et al. [22] found lower values of Cl (3.96L/h) than those presented in this study, while Al-Khofide H et al. [45] found higher values of V (70L).

Bury D et al [23] conducted a study comparing the pharmacokinetics in patients with or without neutropenia, a model was developed with the effect of the neutropenic state and ClCr on vancomycin’s Cl and the effect of fat-free mass in $V_{1}$ and $V_{2}$. Creatinine clearance has been identified as a covariate explaining VAN’s Cl in other models in cancer patients [21,22,24] and total body weight has also been identified as a covariate affecting VAN’s Cl [24].

In order to explore the factors that affect the achievement of VAN pharmacotherapeutic goals in this patient population, artificial neural network (ANN) models were trained; these models presented satisfactory predictive performance values (see Table 3). The ANN models were used to determine the dosing schemes in which the pharmacotherapeutic targets can be achieved and make recommendations.

In our study, it is found that the achievement of the pharmacotherapeutic target of ${\mathrm{AUC}}_{24h}/\mathrm{MIC}\geq 400$ is dependent on the variable MIC. In this way, a very low value of PTA is expected in the case of bacteria with a value MIC of 2mg/L or higher, and doses of more than 3g per day may be required. On the other hand, a MIC value less or equal than 0.5mg/L implies obtaining PTA values close to 1 in all the evaluated dosing schemes. The achievement of a high PTA for ${\mathrm{AUC}}_{24h}/\mathrm{MIC}\geq 400$ can be modulated with changes on daily dose that must be made, considering the level of eGFR presented by an individual. A dose of 1250 mg q12h with an infusion time of 2 h is recommended in individuals with eGFR between 80.4 and $122.4\;\mathrm{mL}/\min/1.73\;m^{2}$. In addition, changes in II or $T_{\inf}$ do not lead to significant differences in the achievement of the target.

On the other hand, the achievement of the target for AUC between 400 and 600mg·h/L is consistently affected by the daily dose and eGFR. It is expected that the daily doses between 2 and 2.5g present a better achievement of the target compared to higher or lower daily doses. eGFR has a relationship directly proportional to the PTA. When the II is greater than 6 h, this variable does not have a significant effect on the PTA. The increase in $T_{\inf}$ has a minimal effect on the achievement of the target when compared to dose changes. The achievement of the $C_{\min}$-based target is not a good surrogate index of the achievement of AUC-based target, since they are not achieved with the same probability in identical dosing regimens. Likewise, this index is affected by other variables such as II or $T_{\inf}$, while variables such as DD and eGFR have little importance in the prediction.

We found very few studies comparing the achievement of PK/PD targets based on AUC with those based on $C_{\min}$ for VAN. In the study by Gatta D. et al. [46], an assessment of the VAN dose was performed in patients with hematological malignancies, and it was found that a dose of 2g/day does not allow reaching the PK/PD targets in patients with creatinine clearance ClCr>60mL/min. It was concluded that patients with ClCr between 60–120 mL/min required doses of 3 g/day, and patients with ClCr>120mL/min required 4g/day in treating infections with *S. aureus* susceptible to VAN [46].

In this study, the reduction of the model was successfully applied through the training of an ANN. The trained model allowed the evaluation of the relationship between controllable and non-controllable variables with the probability of achieving pharmacotherapeutic goals. The decrease in calculation times is beneficial since: (i) it allows the application of mathematical optimization to find the lowest doses that maximize the scope of PK/PD goals, and (ii) it allows a thorough evaluation of the effect of the variables on the outcomes [47]. However, it should be considered that the reduction of the model is limited to the ranges of the variables used in the training of the network [37]. For this study, the effect of the change of the threshold on the PK/PD targets, the effect of the change of the parameters of the population model, or the PTA during the initial phases of treatment, was not studied.

Exposure-related variables (such as daily dose or eGFR) have more impact on achievement of AUC-based PK/PD targets than the one based on $C_{\min}$, which is in turn is affected by variables such as $T_{\inf}$ and II. For this reason, if achievement of the target for $C_{\min}$ in 15–20 mg/L is used as a surrogate of AUC between 400 and 600mg·h/L, it could lead to dosage errors since the first index is relatively non-responsive to changes in the daily dose. The little effect of daily dose on index achievement of $C_{\min}$ could explain the increased incidence of nephrotoxicity by using guided therapeutic drug monitoring with this index compared to AUC [15,16]. This observation further reinforces the need to use current recommendations in VAN therapeutic drug monitoring [6].

The main limitation of this study is related to the small sample size used for the elaboration of the model; however, it should be noted that this is a common limitation in pharmacokinetic studies with extensive designs. Although no formal external evaluation of the model was performed, pharmacokinetic parameters and levels of $C_{\mathrm{PRED}}$ were similar to that reported in previous studies. Another limitation is that the achievement of PK/PD goals was evaluated only at a steady state, and this assumption was used due to simplicity in the specification of dosing regimens.

In conclusion, it was observed that the pharmacokinetics of VAN in patients with post-chemotherapy febrile neutropenia is similar to that reported in other populations. Pharmacokinetic models predict that a 25% increase in daily dose is required compared to a conventional dose of 1g q12h for this population in order to achieve the PK/PD target of ${\mathrm{AUC}}_{24h}/\mathrm{MIC}\geq 400$ on steady state This recommendation changes according to the level of eGFR presented by the individual and outside the range between 80 and $122\mathrm{mL}/\min/1.73\;m^{2}$ further dose adjustments are required.

## Figures and Tables

**Table 1:** Characteristics of patients in the study group.

Variables	Value
Age (years) [Mean (95% CI)]	44 (33.6, 54.4)
Sex (Male/Female)	8 (57.1%)/6(42.9%)
Weight (kg) [Median (IQR)]	60.0 (55.0, 67.0)
Height (cm) [Mean (95% CI)]	163.9 (159.3, 168.4)
Body Surface Area ($m^{2}$) [Median (IQR)]	1.64 (1.55, 1.72)
$S_{\mathrm{CR}}$ ($m^{2}$) [Mean (95% CI)]	0.582 (0.501, 0.663)
eGFR ($\mathrm{mL}/\min/1.73\;m^{2}$) [Mean (95% CI)]	120.3 (109.9, 130.7)
Protein (g/dL) [Mean (95% CI)]	5.59 (5.07, 6.12)
Albumin (g/dL) [Mean (95% CI)]	3.12 (2.84, 3.41)
RAL (/${\mathrm{mm}}^{3}$) [Median (IQR)]	960 (650, 1260)
RAN (/${\mathrm{mm}}^{3}$) [Mean (95% CI)]	264 (187, 341)

**Table 2:** Estimated parameters for the final VAN model.

Parameter	Final Model (RSE%)	Median Bootstrap Value [95% CI]	Shrinkage (%)
Population Parameters			
CL: $\theta_{0}$(L/h)	5.2 (5.94)	5.19 [2.86, 5.85]	
Cl: $\theta_{1}$	0.62 (35.3)	0.58 [0.04, 1.35]	
Q(L/h)	4.99 (21.6)	5.24 [3.50, 27.17]	
$V_{1}\;\left(L\right)$	21.22 (7.28)	20.62 [8.03, 25.48]	
V2(L)	28.28 (13.5)	28.61 [19.28, 312.98]	
Between-subject variability			
$\omega_{\mathrm{CL}}^{2}\;\left(\%\right)$	22.3 (19.4)	21.2 [9.0, 40.5]	3.11
$\omega_{Q}^{2}\;\left(\%\right)$	76.7 (25.9)	68.5 [9.0, 129.0]	−1.03
$\omega_{V1}^{2}\;\left(\%\right)$	22.3 (24.8)	26.4 [8.0, 99.6]	13.4
$\rho_{V_{1}-\mathrm{Cl}}$	0.62 (32.2)	0.61 [−0.24, 0.95]	
Residual variability			
*b*	0.025 (9.5)	0.03 [0.02, 0.04]	19.5

**Table 3:** Predictive performance in trained neural networks for training and validation data generated from the model.

Model	MAE		RMSE	
	Training	Test	Training	Test
Model 1: ANN with one output PTA of ${\mathrm{AUC}}_{24h}/\mathrm{MIC}\geq 400$	0.00189	0.00199	0.00665	0.00697
Model 2: ANN with two outputs: PTA of $C_{\min}$ between 15–20 mg/L and PTA of ${\mathrm{AUC}}_{24h}$ between 400 and 600 mg·h/L	0.0327	0.0313	0.0572	0.0542

## Data Availability

Not applicable.

## Abbreviations

The following abbreviations are used in this manuscript:

ANC	Absolute neutrophil count
ANN	Artificial Neural Network
AKI	Acute Kidney Injury
AUC	Area under the curve
AUC/MIC	Ratio of AUC over MIC
$C_{\min}$	Minimal concentration
$C_{\mathrm{PRED}}$	Predicted concentration
Cl	Clearance
ClCr	Creatinine clearance
DD	Daily dose
GAM	Generalized additive models
eGFR	Estimated glomerular filtration rate
II	Interval between doses
IQR	Interquartile range
MAE	Mean absolute error
MAPE	Mean absolute error percentage
MIC	Minimal Inhibitory Concentration
pcVPC	Prediction-corrected visual predictive check
PTA	Probability of Target Attainment
PK/PD	Pharmacokinetic and Pharmacodynamic
$S_{\mathrm{CR}}$	Serum creatinine
$T_{\inf}$	Infusion time
TVCl	Typical value of clearance
ReLUs	Rectified linear units
RMSE	Root mean quadratic error
V	Volume of distribution
VAN	Vancomycin
